# HIV-1 and IL-1β regulate astrocytic CD38 through mitogen-activated protein kinases and nuclear factor-κB signaling mechanisms

**DOI:** 10.1186/1742-2094-8-145

**Published:** 2011-10-25

**Authors:** Manmeet K Mamik, Sugato Banerjee, Timothy F Walseth, Renee Hirte, Lin Tang, Kathleen Borgmann, Anuja Ghorpade

**Affiliations:** 1 Department of Cell Biology and Anatomy, University of North Texas Health Science Center, 3500 Camp Bowie Blvd, Fort Worth, TX 76107, USA; 2 Department of Pharmacology, University of Minnesota, 6-120 Jackson Hall, 321 Church St. S.E. Minneapolis, MN, USA

## Abstract

**Background:**

Infection with human immunodeficiency virus type-1 (HIV)-1 leads to some form of HIV-1-associated neurocognitive disorders (HAND) in approximately half of the cases. The mechanisms by which astrocytes contribute to HIV-1-associated dementia (HAD), the most severe form of HAND, still remain unresolved. HIV-1-encephalitis (HIVE), a pathological correlate of HAD, affects an estimated 9-11% of the HIV-1-infected population. Our laboratory has previously demonstrated that HIVE brain tissues show significant upregulation of CD38, an enzyme involved in calcium signaling, in astrocytes. We also reported an increase in CD38 expression in interleukin (IL)-1β-activated astrocytes. In the present investigation, we studied regulatory mechanisms of CD38 gene expression in astrocytes activated with HIV-1-relevant stimuli. We also investigated the role of mitogen-activated protein kinases (MAPKs) and nuclear factor (NF)-κB in astrocyte CD38 regulation.

**Methods:**

Cultured human astrocytes were transfected with HIV-1_YU-2 _proviral clone and levels of CD38 mRNA and protein were measured by real-time PCR gene expression assay, western blot analysis and immunostaining. Astrocyte activation by viral transfection was determined by analyzing proinflammatory chemokine levels using ELISA. To evaluate the roles of MAPKs and NF-κB in CD38 regulation, astrocytes were treated with MAPK inhibitors (SB203580, SP600125, U0126), NF-κB interfering peptide (SN50) or transfected with dominant negative IκBα mutant (IκBαM) prior to IL-1β activation. CD38 gene expression and CD38 ADP-ribosyl cyclase activity assays were performed to analyze alterations in CD38 levels and function, respectively.

**Results:**

HIV-1_YU-2_-transfection significantly increased CD38 mRNA and protein expression in astrocytes (p < 0.01) in a dose-dependent manner and induced astrocyte activation. IL-β-activation of HIV-1_YU-2_-transfected astrocytes significantly increased HIV-1 gene expression (p < 0.001). Treatment with MAPK inhibitors or NF-κB inhibitor SN50 abrogated IL-1β-induced CD38 expression and activity in astrocytes without altering basal CD38 levels (p < 0.001). IκBαM transfection also significantly inhibited IL-1β-mediated increases in CD38 expression and activity in astrocytes (p < 0.001).

**Conclusion:**

The present findings demonstrate a direct involvement of HIV-1 and virus-induced proinflammatory stimuli in regulating astrocyte-CD38 levels. HIV-1_YU-2_-transfection effectively induced HIV-1*p*24 protein expression and activated astrocytes to upregulate CCL2, CXCL8 and CD38. In astrocytes, IL-1β-induced increases in CD38 levels were regulated through the MAPK signaling pathway and by the transcription factor NF-κB. Future studies may be directed towards understanding the role of CD38 in response to infection and thus its role in HAND.

## Background

Human immunodeficiency virus (HIV)-1 infection of the central nervous system (CNS) follows soon after initial infection and results in neurocognitive impairment in almost 50% of the infected individuals [1]. The prevalence of these disorders, collectively called HIV-1-associated neurocognitive disorders (HAND), is increasing due to longer life span of infected individuals and poor penetration of anti-retroviral drugs across the blood brain barrier [2]. HIV-1-associated dementia (HAD) constitutes the most severe form of HAND and afflicts 9-11% of the HIV-1-infected individuals even in the era of anti-retroviral therapy [3]. HIV-1-encephalitis (HIVE), the pathological correlate of HAD, is characterized by cytokine/chemokine dysregulation and glial activation [4]. Apart from macrophages and microglia, the astrocytes are implicated as significant contributors to HIV-1 neuropathogenesis [5]. Infected microglia and activated astrocytes contribute to neurotoxicity, which results indirectly from signals exchanged between the two cell types leading to secretion of potential toxic molecules within the CNS, including interleukin (IL)-1β[6]. Astrocytes are in close contact with neurons and are able to sense neuronal activity. Thus, intracellular calcium concentration in astrocytes, mediated by transmitter receptors, is important for determining neuronal activity [7]. Taken together, enzymes involved in calcium signaling are important target molecules for studying mechanisms underlying astrocyte activation and HIV-1 neuropathogenesis.

Human CD38 is a 45 kDa type II, single pass transmembrane glycoprotein expressed by premature hematopoietic cells, lost in mature cells and re-expressed by activated lymphocytes and astrocytes in the brain [8]. Its subcellular localization suggests multiple roles at distinct sites in both neurons and astrocytes. The extracellular domain of CD38 acts as a calcium-mobilizing ectoenzyme that has both adenosine diphosphate (ADP)-ribosyl cyclase and cyclic ADP-ribose (cADPR) hydrolase enzyme activities [9]. cADPR is implicated as a second messenger in neuronal calcium signaling [10]. In HIV-1-infected patients, increased T-cell CD38 expression indicates disease progression, whereas decreased CD38 expression is a good indicator of the effectiveness of anti-retroviral therapy [11]. The three dimensional structure of CD38 shows a peptide region of the molecule to interfere with HIV-1-CD4 receptor interaction, the point of entry for the virus into the cells [12]. This makes the molecule an interesting target for study in HIV-1-associated neurological disorders.

CD38 is upregulated by various cytokines, estrogen and vitamin D3 [13]. Our earlier findings demonstrate that astrocyte CD38 levels are upregulated by interleukin (IL)-1β, and this effect is potentiated by HIV-1 envelope glycoprotein (gp120) [14]. This leads to a rise in intracellular calcium concentration [14] and disrupts glutamate transport by astrocytes [15], eventually resulting in excitotoxic neuronal damage [16].

HIV-1 infection of astrocytes is restricted and nonproductive (as reviewed in [17]). This makes it difficult to study direct effects of the virus on astrocyte biology. To overcome the restricted HIV-1 entry into the astrocytes, in the current study, we employed a high-efficiency transfection technique to directly deliver HIV-1_YU-2 _plasmid into astrocytes. This allowed us to mimic direct effects of the HIV-1 gene expression and replication alone on astrocyte activation and CD38 regulation. Our laboratory has previously shown increased astrocyte CD38 expression in HIV-1-infected human brain tissues [18]. The CD38 gene, located on chromosome 4 in humans, is regulated by physiological stimuli such as tumor necrosis factor (TNF)-α, IL-1β and interferon-γ, which are produced by activated astrocytes [19-21]. The 5' upstream region of the CD38 gene has absence of TATA and CAAT boxes and presence of various binding sites for transcription factors such as activator protein-1 and nuclear factor (NF)-κB [22]. The principal components in the signaling cascades resulting in activation of NF-κB upon various stimuli are the mitogen activated protein kinases (MAPKs). MAPKs are a family of serine threonine kinases comprising of extracellular signal regulated kinase (ERK), p38 kinases (p38Ks) and c-Jun N-terminal kinases (JNK), and can regulate various aspects of astrocyte biology [23-25]. IL-1β can mediate activation of ERK 1/2, p38Ks and JNK phosphorylation in mixed glial cells that may play an important role during neuroinflammation [26]. After activation, MAPKs can regulate gene expression at transcriptional, translational and post-translational levels. IL-1β is an HIV-1-relevant mediator of inflammation [27] and regulates NF-κB in astrocytes [28]. We have previously shown CD38 upregulation in IL-1β-activated astrocytes [14].

In the present study, we hypothesized that the MAPK signaling system participates in the upregulation of CD38 gene expression in response to HIV-1-relevant stimuli such as HIV-1_YU-2 _and IL-1β, via NF-κB transcription factor. We show that induced HIV-1 gene expression and replication in astrocytes, and stimulation with IL-1β, increase the level of CD38 expression via a MAPK-NF-κB dependent mechanism. Thus, the data presented here provide important clues on the contribution of CD38 in astrocyte-mediated neuroinflammatory processes involved in neurodegenerative disorders such as HAND.

## Methods

### Isolation and cultivation of primary human astrocytes

Human astrocytes were isolated from elective abortus specimens procured in full compliance with the ethical guidelines of the NIH, the University of Nebraska Medical Center, University of Washington and North Texas Health Science Center, as previously described [29]. Briefly, brain tissues were dissected and mechanically dissociated. Cell suspensions were centrifuged, suspended in media, and plated at a density of 20×10^6 ^cells/150 cm^2^. The adherent astrocytes were treated with trypsin and cultured under similar conditions to enhance the purity of replicating astroglial cells. The astrocyte preparations were routinely >99% pure as measured by immunocytochemistry staining for glial fibrillary acidic protein (GFAP) and microglial marker CD68 to rule out any microglial contamination and contribution of microglia in inflammatory responses.

### RNA extraction and gene expression analysis

RNA was isolated (as described in [30]) from astrocytes treated as described in subsequent sections and gene expression was assayed by real-time PCR. TaqMan 5' nuclease real-time PCR was performed using an ABI Prism 7900 sequence detection system (Applied Biosystems Inc., Foster City, CA). Commercially available TaqMan^® ^Gene Expression Assays were used to measure CD38 and GAPDH mRNA levels (Applied Biosystems). GAPDH, a ubiquitously expressed housekeeping gene, was used as an internal normalizing control. The 30 μl reactions were carried out at 48°C for 30 min, 95°C for 10 min, followed by 40 cycles of 95°C for 15 s and 60°C for 1 min in 96-well optical, real-time PCR plates.

### HIV-1_YU-2_- and IκBαmutant-transfection of astrocytes

Primary human astrocytes were transfected with HIV-1_YU-2 _(obtained from the AIDS Research and Reference Reagent Program, Division of AIDS, NIAID, NIH: pYU-2 from Dr. Beatrice Hahn and Dr. George Shaw [31]) or IκBα mutant (IκBαM, addgene plasmid 12330, deposited by Dr. Inder M Verma [32]) plasmids using the Amaxa Rat Astrocyte Nucleofector kit (Lonza Walkersville Inc., Walkersville, MD, USA). Briefly, astrocytes were suspended in nucleofector solution and HIV-1_YU-2 _plasmid (0.2, 0.3, 0.4, 0.8 μg/1.5 million cells) or IκBβM plasmid (40 μg/8 million cells) and transfected using a Nucleofector/Shuttle (Lonza) device. To assess transfection efficiency, seven images were taken from multiple wells of HIV-1_YU-2_-transfected astroctyes and the number of GFAP positive and HIV-1*p24 *positive cells in each image were counted independently. The number of cells positive for both markers was then calculated as a percentage. Transfected cells were supplemented with astrocyte media and incubated for 30 min at 37°C prior to plating. Twelve to 24 h post-plating, cells were washed and serum-free astrocyte media was added with or without IL-1β20 ng/ml) for 8 h to 7 d.

### Astrocyte treatment and activation

Primary astrocytes were treated with or without MAPK inhibitors SB 203580 (20 μM), SP 600125 (20 μM) and U0126 (20 μM, Sigma Aldrich Inc., St Louis, MO) or with a peptide inhibitor of NF-κB translocation into the nucleus, SN50 (18 μM), or corresponding control mutant peptide, SN50(M) (18 μM, Sigma), for 1 h prior to IL-1β-activation (20 ng/ml) for 8 h in serum-free astrocyte media, as previously described [14,18]. This dose is well within the range of 5-100 ng/ml currently used by many other groups to activate astrocytes [33] and levels induced in animal models [34,35].

### Measurement of proteins

Viral gene expression in astrocytes was determined by measuring viral capsid protein HIV-1*p24 *levels by immunocytochemistry 5 days post-transfection. Mock and HIV-1_YU-2_-transfected astrocytes were immunolabeled as previously described [29] with HIV-1*p24 *antibody (1:10, Dako Corp Inc., Carpinteria, CA), GFAP antibody (1:1000, Dako) and/or CD38 antibody (1:100, Novo Castra, United Kingdom) to evaluate viral expression and CD38 expression. Protein expression in whole cell or culture supernatant was also quantified by HIV-1*p24 *ELISA (Perkin Elmer Inc, Waltham, MA), CCL2 and CXCL8 ELISA (R&D systems Inc., Minneapolis, MN) at 1, 2, 4 and 5 days after HIV-1_YU-2_-transfection.

### Determination of ADP-ribosyl cyclase activity

The ADP-ribosyl cyclase activity of primary astrocyte lysates was quantified using a fluorescent cycling assay that measures the production of nicotinamide adenine dinucleotide (NAD) from cADPR and nicotinamide as described in [36]. Briefly, cells were harvested in Tris-sucrose buffer (pH 7.2) with protease inhibitors. Cell lysates containing 5 μg of total protein were incubated with 10 mM or without nicotinamide in the presence of 0.45 mM cADPR. NAD was quantified by a cycling reaction that generates a fluorescent product. The fluorescence was quantified (excitation at 544 nm and emission at 590 nm) in a FLUOstar Galaxy fluorometer (BMG Biotechnologies, Durham, NC, USA), and the rate of emission of fluorescence was calculated. A standard curve generated from known NAD standards was used to calculate the quantity of NAD generated in experimental reverse cyclase reactions. The ADP-ribosyl cyclase activity is expressed in femtomoles of NAD per minute per milligram of total protein.

### Western blot analysis

Equal amounts of protein samples (25 μg) were boiled with 1X Laemmli sample buffer for 5-10 minutes, resolved by 10% sodium dodecylsulfatepolyacrylamide gel electrophoresis and subsequently transferred to a nitrocellulose membrane using i-Blot (Invitrogen, Carlsbad, CA, USA). The membrane was incubated with anti-human mouse CD38 antibody (1:250, BD Biosciences) overnight at 4°C, washed and then incubated with anti-mouse goat antibody IgG conjugated to horseradish peroxidase (1:5,000, Bio-Rad) for 2 h at room temperature. The membrane was then developed with SuperSignal west femto substrate (Thermo Fisher Scientific, Rockford, IL) in an Flourochem HD2 Imager (ProteinSimple, Inc. Santa Clara, CA). α-tubulin (1:1,000, Cell Signaling) immunoblotting was used as a loading control.

### Determination of astrocyte metabolic activity

Following experimental manipulations described above, five percent (3-(4,5-dimethylthiazol-2-yl)-2,5-diphenyltetrazolium bromide (MTT) reagent in astrocyte medium was added to astrocytes and incubated for 20-45 min at 37 °C. MTT is metabolically reduced to purple formazan crystals by living cells. The MTT solution was removed and crystals were dissolved in DMSO for 15 min with gentle agitation. The absorbance of the DMSO/crystal solution was assayed for absorbance at 490 nm in a Spectromax M5 microplate reader (Molecular Devices, Sunnyvale, CA).

### Statistical analyses

Statistical analyses were carried out using GraphPad Prism 5.0 software, with one-way analysis of variance (ANOVA) and Newman-Keuls post-test for multiple comparisons. Significance was set at p < 0.05 and data represents means +/- standard error of the mean (S.E.M.). Data presented is representative of a minimum of three independent experiments with two or more independent donors.

## Results

### HIV-1_YU-2_-transfection enhances CD38 expression in astrocytes

**Figure 1:**
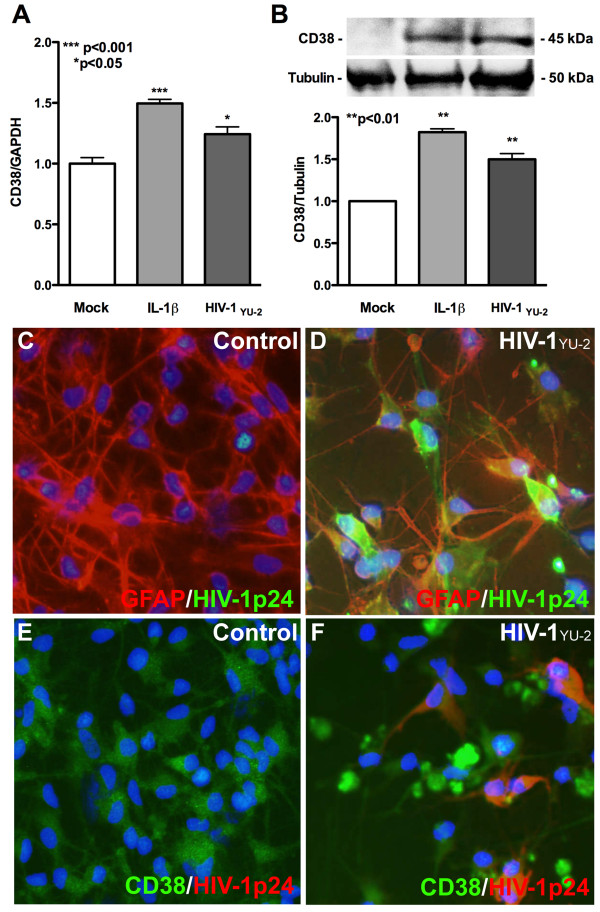
**Astrocyte HIV-1_YU-2_-transfection increases CD38 expression**. Cultured human astrocytes were transfected with HIV-1_YU-2 _plasmid and mock-transfected controls were maintained in parallel. RNA was isolated 24 h post-transfection and assayed for CD38 levels. **(A) **By real-time-PCR assay, significantly higher CD38 mRNA levels were detected in HIV-1_YU-2_-transfected astrocytes as compared to mock controls (p < 0.05). **(B) **HIV-1_YU-2_-transfected astrocytes showed significantly increased CD38 protein levels as compared to mock controls when assayed by western blot and densitometry analyses of whole cell protein lysates (p < 0.01), 5 days post-transfection. IL-1β (20 ng/ml)-treated astrocytes were maintained as positive controls for increase in CD38 mRNA and protein levels. Graph shows representative data from two independent donors. Expression of CD38 and HIV-1p24 was measured by immunocytochemistry 5 days post-transfection **(C-F)**. Nuclei were stained in blue by DAPI in all panels. **(C) **Mock, GFAP-positive astrocytes (red) with no HIV-1p24 expression. **(D) **Co-localization (yellow) of GFAP (green) and HIV-1p24 (red) in HIV-1_YU-2_-transfected astrocytes. Transfection efficiency was routinely 90% as assessed by GFAP, HIV-1p24 co-localization (data not shown) **(E) **Basal CD38 expression (green) and no HIV-1p24 expression in mock control. **(F) **Increased CD38 expression (green) and co-localization (yellow) with HIV-1p24 (red) in HIV-1_YU-2_-transfected astrocytes. Original magnification x200.

**Figure 2:**
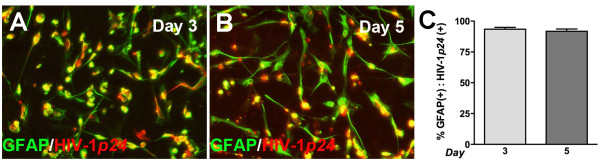
**Transfection efficiency of HIV-1_YU-2 _gene expression plasmid in cultured human astrocytes**. To assess the transfection efficiency of cultured human astrocytes with HIV-1_YU-2_, astrocytes were transfected with 0.3 μg/1.5 million cells using the Amaxa Rat Astrocyte Nucleofector kit and plated in 48 well tissue culture plates at 1_10_^5 ^cells/well. Three and five days post transfection cells were fixed and immunocytochemically labeled with an astrocyte marker, GFAP (green) and HIV-1*p24 *(red). Seven images were taken from multiple wells of HIV-1_YU-2_-transfected astroctyes and the number of GFAP positive and HIV-1*p24 *positive cells in each image were counted independently. The number of cells positive for both markers was then calculated as a percentage. **(A) **Day three representative image (200x, original magnification), **(B) **Day five representative image (200x, original magnification) **(C) **Percent double positive cells following analysis of all images on both day 3 and day 5. An average 90% transfection efficient was achieved with the HIV-1_YU-2 _gene expression plasmid by this method in cultured human astroctyes.

Astrocytes lack surface CD4; therefore virus can infect only a small fraction of cells *in vitro *and *in vivo *[37]. We have shown that IL-1 μalone or in combination with HIV-1gp120, leads to increased CD38 expression and function in cultured human astrocytes [18]. In this study, we employed astrocyte transfection with HIV-1 proviral DNA to bypass receptor restriction and enable intracellular entry of the HIV-1_YU-2 _viral genome, a brain-derived isolate. To determine the role of HIV-1 in modulating astrocyte CD38 levels, we transfected astrocytes with HIV-1_YU-2 _gene expression plasmid and measured CD38 mRNA and protein levels. One day post-transfection, CD38 mRNA levels increased significantly in HIV-1_YU-2_-transfected cells as compared to mock (Figure 1A, p < 0.01). In addition, to assess CD38 expression at translational level, whole cell protein lysates were analyzed by western blot and densitometry analyses five days post-transfection. HIV-1_YU-2_-transfected astrocytes showed significantly increased CD38 protein levels (normalized to α-tubulin) as compared to mock. Astrocytes were also treated with IL-1β (20 ng/ml) to serve as positive control for increased CD38 protein levels. (Figure 1B, p < 0.01). In parallel, astrocytes were immunoassayed for CD38 and HIV-1*p24, *five days post-transfection. HIV-1*p24 *antigen-expression was evident in HIV-1_YU-2_-transfected astrocytes by co-localized immunostaining (yellow) of HIV-1*p24 *(green) with GFAP (red; Figure 1D), but not in mock (Figure 1C). HIV-1_YU-2_-transfected astrocytes showed more intense CD38 staining (Figure 1F) as compared to mock (Figure 1E). Furthermore, CD38 (green) co-localized with HIV-1*p24 *(red, Figure 1F) in HIV-1_YU-2_-transfected astrocytes. Taken together, HIV-1_YU-2_-transfection significantly increased CD38 RNA and protein levels in astrocytes. Transfection efficiency for the HIV-1_YU-2 _plasmid was routinely 90% as assessed by GFAP, HIV-1*p24 *co-staining (Figure 2).

### CD38 mRNA expression corresponds with viral gene expression in HIV-1_YU-2_-transfected astrocytes

**Figure 3:**
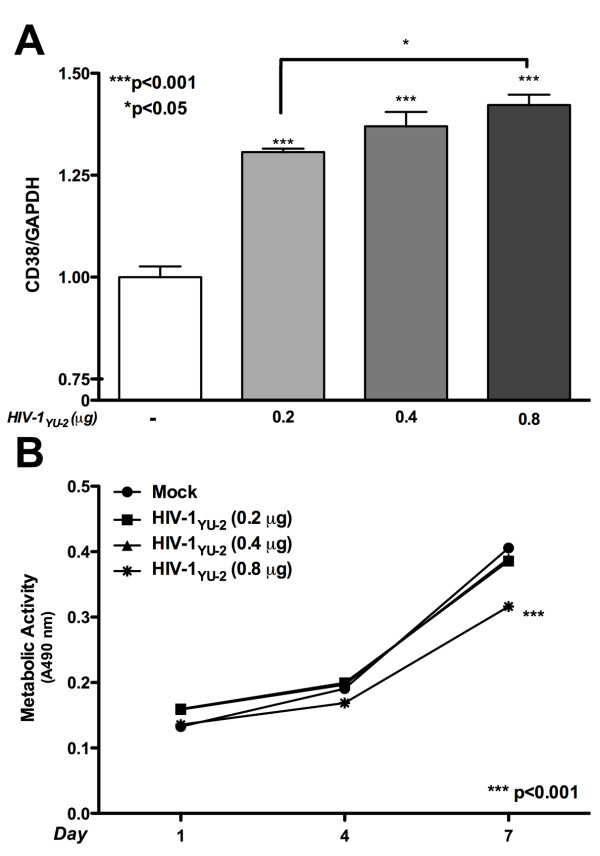
**CD38 mRNA expression corresponds with viral gene expression in HIV-1_YU-2 _transfected astrocytes**. Astrocytes were transfected with a dose of HIV-1_YU-2 _plasmid 0.2, 0.4 and 0.8 μg/1.5 million cells and assayed for CD38 expression and cell viability. **(A) **Twenty four hours post-HIV-1_YU-2_-transfection, CD38 expression increased significantly in a dose-dependant manner a measured by RT-PCR (p < 0.001). **(B) **Metabolic activity levels were unchanged by HIV-1_YU-2 _transfection at 0.2 and 0.4 μg as compared to mock. However, at 0.8 μg metabolic activity was significantly reduced at 7 day (p < 0.001). In subsequent experiments astrocytes were transfected with 0.3 μg and mRNA and protein levels were assayed at day five, to ensure viability of HIV-1_YU-2_-transfected astrocytes was not compromised during the experimental time course.

CD38 expression increased in a dose-dependant manner (0.2, 0.4 and 0.8 μg/1.5 million cells) in HIV-1_YU-2_-transfected astrocytes as measured by RT-PCR at day 1 (Figure 3A, p < 0.001) which corresponded with increased HIV-1*p24 *expression (data not shown). Metabolic activity levels were unchanged by HIV-1_YU-2 _transfection (0.2 and 0.4 μg/1.5 million cells) as compared to mock. However, at 0.8 μg/1.5 million cells metabolic activity was significantly reduced at 7 day (Figure 3B, p < 0.001). In subsequent experiments astrocytes were transfected with 0.3 μg HIV-1_YU-2 _plasmid and mRNA and protein levels were assayed at day five, to ensure viability of HIV-1_YU-2_-transfected astrocytes was not compromised during the experimental time course.

### HIV-1_YU-2_-transfection leads to HIV-1-associated protein expression and astrocyte activation

**Figure 4:**
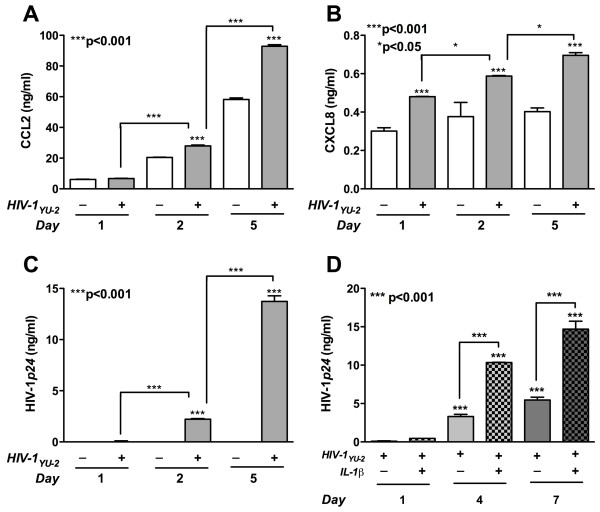
**HIV-1_YU-2_-transfected astrocytes have enhanced levels of proinflammatory chemokines in a dose and time dependent manner**. Following HIV-1_YU-2_-transfection, HIV-1p24, CCL2 and CXCL8 levels were assayed by ELISA. **(A) **HIV-1_YU-2_-transfection significantly increased culture supernatant cumulative CCL2 levels by 2 and 5 days as compared to both respective mock controls and day 1, HIV-1_YU-2_-transfected astrocytes (p < 0.001). **(B) **Astrocyte CXCL8 levels in the supernatants also increased significantly upon HIV-1_YU-2_-transfection as compared to mock controls (p < 0.001). CXCL8 levels in HIV-1_YU-2_-transfected astrocytes continued to increase significantly as compared to the previous day, over five days post-transfection (p < 0.05). **(C) **Cumulative HIV-1p24 levels in total cell extracts were assayed on 1, 2 and 5 days post-transfection to quantify viral protein expression. HIV-1_YU-2_-transfection significantly increased HIV-1p24 levels as compared to mock on day 2 and day 5 (p < 0.001). **(D) **In HIV-1_YU-2_-tranfected astrocytes treated with and without IL-1β a significant concomitant increase in HIV-1p24 expression was observed at both 4 and 7 days (p < 0.001).

Since production of chemokines CCL2 and CXCL8 is associated with astrocyte activation [38], we measured their levels in culture supernatants following HIV-1_YU-2_-transfection at different time points. Viral transfection resulted in activation of astrocytes as evident from gradual increases in the production of CCL2 and CXCL8 from days one through five. CCL2 and CXCL8 levels were significantly increased in HIV-1_YU-2_-transfected astrocytes by days two and five as compared to respective mock controls and to day 1 HIV-1_YU-2_-transfected astrocytes (Figure 4A-B, p < 0.001, respectively). To ensure successful viral protein expression post-transfection, HIV-1*p24 *levels were determined by ELISA. HIV-1*p24 *levels increased ~two-fold by day 2 and ~13-fold by day 5 (Figure 4C, p < 0.001). These observations are consistent with previous works, showing astrocyte activation five days post-infection with vesicular stomatitis virus pseudotyped HIV-1 astrocyte infection [39]. IL-1β along with TNF-α is known to reactivate latent or non-productive HIV-1 infection of astrocytes [40] in an NF-κB dependent manner [41]. In this HIV-1 gene expression model, IL-1β-activation significantly increased HIV-1*p24 *expression in HIV-1_YU-2_-tranfected cells at both 4 and 7 days (Figure 4D, p < 0.001). Since IL-1β is upregulated in brain macrophages and microglia and a potent activator of CD38, subsequent signaling studies were performed in the context of IL-1β-induced CD38 expression. Human astrocytes with HIV-1_YU-2_-expressing plasmid become activated and demonstrate increased CD38 levels *in vitro*. These data demonstrate that HIV-1_YU-2 _expression in human astrocytes leads to enhanced CD38 expression and proinflammatory chemokine production.

### MAPKs regulate IL-1β-induced activation of CD38 expression

**Figure 5:**
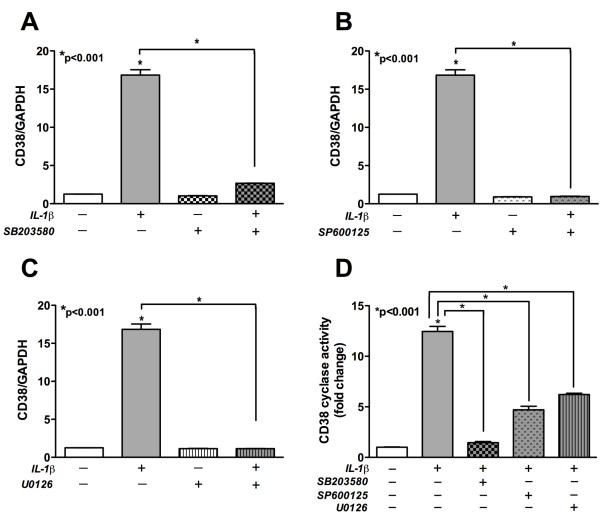
**MAPKs regulated CD38 expression and function in IL-1β-activated astrocytes**. Following 1 hour pre-treatment with various MAPK inhibitors, primary astrocytes were activated with IL-1β, 20 ng/ml. Untreated controls were maintained in parallel. CD38 mRNA levels were measured using real-time PCR while CD38 activity, as a measure of CD38 function, was analyzed using cADPR cyclase assay. As expected, IL-1β alone significantly increased CD38 expression and activity as compared to untreated controls (p < 0.001, all panels). Basal CD38 expression was unchanged (p > 0.05, A-C) by inhibitor pre-treatment. The IL-1β-induced increase in CD38 mRNA expression was abrogated by pre-treatment with inhibitors specific to **(A) **p38, SB203580 (p < 0.001) **(B) **JNK, SP600125 (p < 0.001) and **(C) **ERK, U0126 (p < 0.001). **(D) **Pre-treatment with each of the MAPK inhibitors significantly inhibited the IL-1β-mediated CD38 cADPR cyclase activity in astrocytes (p < 0.001).

To investigate the role of MAPKs (p38Ks, JNK and ERK) in regulating CD38 expression in astrocytes, a panel of pharmacological inhibitors targeting MAPKs was employed. IL-1β an HIV-1-relevant inflammatory mediator [27,42,43], has been shown to activate ERK, p38Ks and JNK phosphorylation in cultured human astrocytes [26,44]. While inhibition of individual MAPKs (p38Ks, JNK, or ERK) did not significantly reduce (p > 0.05) basal CD38 mRNA expression, each of their respective blockers (p38K: SB203580, Figure 5A; JNK: SP600125, Figure 5B and ERK: U0126, Figure 5C), significantly abrogated the induction of CD38 expression by IL-β (p < 0.001). Thus, activation of p38Ks, JNK and ERK MAPKs likely contribute to increased CD38 mRNA expression in IL-1β-activated astrocytes. The ADP-ribosyl cyclase assay, as a measure of CD38 function, showed significant reduction in IL-1β-induced CD38 ADP-ribosyl cyclase activity upon inhibition of p38Ks, JNK and ERK with their respective pharmacological blockers (Figure 5D, p < 0.001). Thus, we conclude that JNK, p38Ks and ERK are each involved in the modulation of CD38 expression and function in IL-1β-activated human astrocytes.

### CD38 expression and function in IL-1β-activated astrocytes is NF-κB dependent

**Figure 6:**
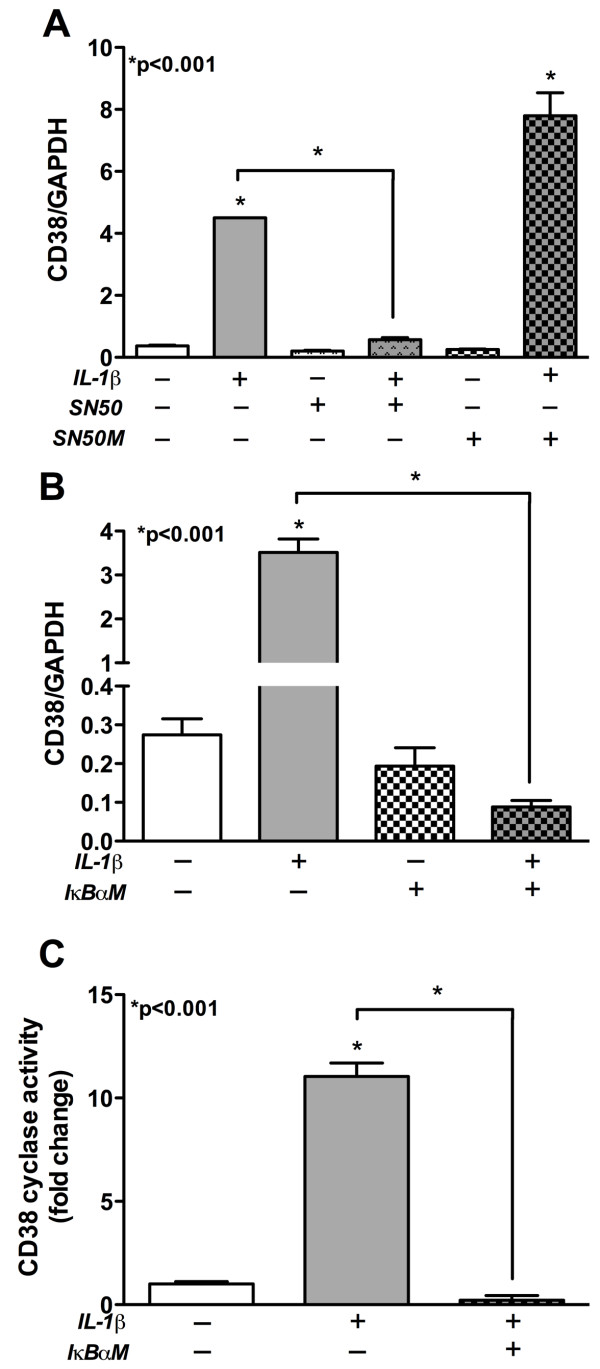
**NF-κB regulates CD38 in IL-1β-activated astrocytes**. Cultured human astrocytes were activated with and without IL-1β in the presence or absence of NF-κB inhibitors, SN50 or IκBαM. CD38 mRNA expression was measured using real-time PCR while CD38 activity was measured using cADPR cyclase assay. **(A) **Pre-treatment of IL-1β-activated astrocytes with NF-κB blocker, SN50, showed significantly lower CD38 mRNA levels as compared to IL-1β-activated astrocytes (p < 0.001). SN50M treatment, however, did not affect IL-1β-responses in astrocytes. **(B) **IκBαM-transfection abrogated the IL-1β-mediated increase in CD38 mRNA levels (p < 0.001) without affecting basal CD38 levels in untreated astrocytes (p > 0.05). **(C) **IκBαM-transfected, IL-1β-activated astrocytes showed a significant reduction (p < 0.001) in CD38 cyclase activity compared to mock-transfected, IL-1β-activated astrocytes.

NF-κB is one of the major mediators of IL-1β signaling in primary human astrocytes [45]. To determine the role of NF-κB in IL-1β-mediated CD38 regulation, cultured astrocytes were pre-treated with a peptide inhibitor of NF-κB translocation into the nucleus, SN50 (18 μM), or non-inhibiting control, SN50M (18 μM). Cells were then activated with IL-1β, 20 ng/ml, for 8 h. SN50 treatment significantly inhibited the IL-1β-induced increase in CD38 expression as compared to IL-1β alone (Figure 6A, p < 0.001). As expected, the control peptide SN50M did not inhibit the IL-1β-mediated increase in CD38 levels (Figure 6A). To further confirm the role of NF-κB in IL-1β-mediated CD38 expression, primary astrocytes were transfected with IκBαM and then activated with IL-1β for 8 h. IκBαM prevents the phosphorylation and subsequent displacement of IκBαM from the NF-κB complex, thus inhibiting NF-κB activity. IκBαM transfection abrogated the IL-1β-mediated increase in CD38 expression as compared to mock, and IL-1β-activated cells (Figure 6B, p < 0.001). Basal CD38 expression in IκBαM-transfected cells remained unaffected. To further confirm the role of NF-κB regulation of CD38 function, we assayed CD38 ADP-ribosyl cyclase activity in whole cell lysates from transfected astrocytes. As expected, IκBαM-transfected astrocytes had negligible CD38 cyclase activity indicating that a molecular block in the NF-κB pathway abrogated CD38 function (Figure 6C, p < 0.001). Thus, we conclude that NF-κB is a significant regulator of IL-1β-mediated increase in CD38 mRNA expression and activity in astrocytes.

## Discussion

In a previous study, our laboratory reported increased CD38 expression in HIVE brains, which co-localized with astrocytes in areas of inflammation [18]. The study established an important role for CD38 in modulating astrocyte neuroinflammatory responses. Here, we extend our analyses by investigating molecular mechanisms and signaling pathways responsible for CD38 modulation in astrocytes. In the present study, we show a direct upregulation of astrocyte-CD38 mediated by HIV-1. Transfection of astrocytes with HIV-1_YU-2 _gene expression plasmid not only increased CD38 mRNA and protein levels but also led to activation of astrocytes, as evident by an increase in production of chemokines CXCL8 and CCL2. *In vivo*, the increased CCL2 is thought to assist in attracting monocytes across the blood brain barrier. It is also implicated that proinflammatory chemokine CCL2 appears in brain soon after the virus enters the CNS [46]. The results suggest that chemokines produced by a limited number of infected astrocytes may lead to immune cell recruitment and subsequent activation of non-infected astrocytes, thereby further upregulating astrocyte-CD38 as a whole. As we previously reported, increased CD38 enzyme activity leads to increased cADPR levels and a corresponding rise in intracellular calcium flux in activated astrocytes [14,18]. The CD38/cADPR system is thought to initiate astrocyte to neuron calcium signaling, which then leads to increased release of neuromodulators from glial cells [47]. Imbalance in calcium signaling may eventually lead to neuronal dysfunction [48].

Astrocytes may not be capable of *de novo *viral replication, but HIV-1-infected astrocytes can transmit the virus to CD4+ cells. Viral particles are released from astrocytes without reverse transcription. While this mode of infection does not increase viral load; it can, however, lead to viral persistence and spreading throughout the CNS [49,50]. Since astrogliosis is a prominent feature of early CNS HIV-1 infection [51,52], astrocytes are likely to be neuroprotective at the early phase of infection. However, dysfunction of astrocytes during chronic HIV-1 CNS infection and immune activation may lead to neurotoxicity [5,39,53]. The precise functional consequences of astrocyte infection and/or activation by HIV-1 remain unclear. Thus, using the model system of transfecting astrocytes with HIV-1 plasmid, we may be able to understand the direct effects of the viral gene expression on astrocyte function and their final impact on neurotoxicity during HIV-1-CNS infection.

Increased IL-1β expression has not been reported in astrocytes in response to various HIV-1 proteins or HIV-1 gene expression and replication models [54-56]. However, IL-1β is elevated in the brain tissues of patients infected with HIV-1 [52], is upregulated and secreted by infected/activated immune cells in the proinflammatory setting of HIV-1 infection [27], and induction of the IL-1β autocrine loop leads to further production of IL-1β and other cytokines [57]. IL-1β along with TNF-α is also known to reactivate latent or non-production HIV-1 infection of astrocytes [40] in an NF-κB dependent manner [41]. Therefore, subsequent signaling studies were performed in the context of IL-1β-induced CD38 expression.

We evaluated the role of transcription factor NF-κB in CD38 regulation. Our study showed that pretreatment of the astrocytes with SN50, a cell permeable peptide inhibitor of NF-κB, blocked the expected CD38 upregulation seen upon IL-1β-activation. This finding strongly emphasized that IL-1β-mediated gene upregulation involved the transcription factor NF-κB. This was further supported by attenuated CD38 expression and enzyme activity following transient transfection of astrocytes with IκBαM, which impeded NF-κB activation. Understanding the regulation of this signaling pathway during neuroinflammatory conditions like HIVE may have important therapeutic implications. The transcription factor NF-κB is a crucial mediator in the IL-1β signaling pathway and acts as a major driving force behind the induction of cytokines, chemokines and adhesion molecules by astrocytes; also important mediators of inflammation during HIVE [58]. Following stimulation, the duration of NF-κB activation may be transient or persistent, depending on the cellular stimulus and cell type. Interestingly, it has been shown that stimulation with IL-1β may result in prolonged NF-κB activation, thus suggesting its implication in neuroinflammation associated with HIVE [59]. Thus, taken together, these findings suggest that NF-κB is one of the major regulators of CD38 expression and enzyme activity in activated astrocytes.

We also investigated the involvement of MAPK in CD38 regulation, since NF-κB is downstream transcription factor in MAPK signaling cascade. Emerging evidence suggests that MAPK signaling pathway may play an important role in activated glia-induced neuronal malfunction [60]. MAPKs are important in the transduction of extracellular signals into cellular responses. When activated, these kinases can phosphorylate both cytosolic and nuclear target proteins resulting in the activation of transcription factors and ultimately the regulation of gene expression [25]. IL-1β is known to increase the activation of p38Ks, JNK and ERK MAPKs in primary astrocytes [26,44]. We inhibited the activation of each MAPK pathway independently and showed significant decreases in CD38 expression in IL-1β-activated astrocytes. The IL-1β-induced ADP-ribosyl cyclase activity of CD38 was also significantly reduced by inhibition of each of the p38Ks, JNK and ERK pathways. It should be noted that inhibition of each individual signaling pathway alone, produced robust downregulation in CD38 expression and cyclase activity in IL-1β-activated astrocytes. It is therefore reasonable to assume equal importance of all three MAPK pathways in CD38 regulation. Importantly, the MAPK inhibitors did not affect basal CD38 levels in non-activated astrocytes. Thus, taken together these results suggest that MAPKs regulate IL-1β-induced CD38 levels in astrocytes, either directly or indirectly, through NF-κB. Both p38Ks and JNK have been reported to mediate neuronal damage primarily by glial activation [61]. The activation of p38Ks plays an important role in developing HIV-1 envelope protein gp120-mediated cytotoxicity of human brain microvascular endothelial cells [62]. MAPK activation can lead to nitric oxide production and cytokine release in glial cells, thus exacerbating the neuroinflammatory milieu during neurodegenerative disorders including HIVE [63,64].

It is known that HIV-1 can activate p38Ks, ERK and JNK MAPK cascades, while HIV-1-transactivator may induce both NF-κB and p38Ks, JNK MAPK pathways [65,66] in astrocytes. This may eventually lead to release of glutamate and pro-inflammatory cytokines from glial cells, thus contributing to neurodegeneration during HAD [67]. HIV-1gp120 may also activate MAPKs in neurons [68]. Activation of the NF-κB and MAPK signaling may lead to activation of nitric oxide synthase which can result in release of nitric oxide in both human and rat astrocytes and in C6 glioma cells [69,70]. It has been reported previously that NF-κB activation may lead to release of reactive oxygen species, which in turn regulate inducible nitric oxide synthase expression in astrocytes (as reviewed in [71]). Thus, it will be interesting to understand how modulation of CD38 participates in the release of inducible nitric oxide synthase in IL-1β-activated astrocytes.

It is now well established that activated astrocytes release several inflammatory cytokines and chemokines including IL-1β, IL-6, TNF-α, CCL2 and CXCL8 (reviewed in [72,73]), which are thought to contribute to inflammation associated with HIVE [74]. We have previously demonstrated that the proinflammatory cytokine IL-1β upregulates Fas ligand in astrocytes, which induces apoptosis in neurons [45,53], and that IL-1β-mediated production of CCL2 and CXCL8 is partially regulated by CD38 [18]. Autocrine production of IL-1β can enhance a number of other signaling molecules downstream of the IL-1β signaling cascade [75]. However, we have also shown CD38 expression is independent of the IL-1β-autocrine loop in astrocytes [18]. Therefore, regulation of CD38 in astrocytes is net effect of a complex mechanism.

## Conclusions

Our findings compliment our previous studies and propose a regulatory mechanism for CD38 gene expression in astrocytes during neuroinflammation. IL-1β-induced CD38 upregulation is likely mediated by activation of JNK, p38Ks and ERK MAPK signaling pathways through the downstream transcription factor NF-κB. With the efficient transfection of HIV-1_YU-2 _into astrocytes, we provide evidence that HIV-1 gene expression and replication directly increases CD38 levels in astrocytes. De Flora's group previously demonstrated that increased calcium by CD38/cADPR system may lead to release of glutamate by astrocytes [76]. Excessive exposure to the neurotransmitter glutamate has been implicated as a key factor contributing to neuronal injury and death in HIVE [76,77]. Thus, our current findings contribute towards understanding the role of transcription factors and signaling molecules regulating CD38 levels during neuroinflammatory conditions. Since CD38 is implicated in neuroinflammation, a detailed understanding of its regulatory mechanisms may help in developing means to modulate CD38 levels, consequently controlling neuroinflammation associated with HIV-1-CNS infection.

## Abbreviations

Human immunodeficiency virus (HIV)-1, central nervous system (CNS), mitogen activated protein kinases (MAPKs), HIV-1-associated neurocognitive disorders (HAND), HIV-1-associated dementia (HAD), HIV-1-encephalitis (HIVE), adenosine diphosphate (ADP), cyclic ADP-ribose (cADPR), interleukin (IL), nuclear factor (NF)-κB, mitogen activated protein kinases (MAPKs), extracellular signal regulated kinase (ERK), p38 kinases (p38Ks), c-Jun N-terminal kinases (JNK), glial fibrillary acidic protein (GFAP), IκBα dominant negative mutant (IκBαM), nicotinamide adenine dinucleotide (NAD), non-inhibiting mutant peptide, SN50(M), tumor necrosis factor (TNF)

## Competing interests

The authors have no conflicts of interest to disclose that could have inappropriately influenced, or be perceived to influence, their work.

## Authors' contributions

AG designed research strategy and experiments. TFW performed and advised on the cADPR activity assay data. MKM, SB, TFW, RH, LT and KB performed experiments. MKM and SB drafted and revised the manuscript. All authors read, edited and approved the final manuscript.
